# The immunopathological spectrum of COVID-19: from cytokine storm to autoimmunity—a systematic review

**DOI:** 10.3389/fmed.2026.1864452

**Published:** 2026-06-10

**Authors:** Harika Öykü Dinç, Suat Saribaş, Bekir Kocazeybek

**Affiliations:** 1 Department of Medical Microbiology, Faculty of Medicine, Üsküdar University, Istanbul, Türkiye; 2 Department of Medical Microbiology, Faculty of Cerrahpaşa Medicine, Istanbul University-Cerrahpaşa, Istanbul, Türkiye

**Keywords:** COVID-19, cytokine storm, endothelial dysfunction, immunopathology, SARS-CoV-2, vaccines

## Abstract

**Introduction:**

This systematic review aimed to provide a comprehensive evaluation of the immunopathological mechanisms associated with SARS-CoV-2 infection and COVID-19 vaccination based on current evidence. Particular emphasis was placed on cytokine storm, endothelial dysfunction, complement activation, and autoimmune processes in COVID-19 pathogenesis.

**Methods:**

The study was conducted as a systematic literature review in accordance with PRISMA guidelines. English-language research articles published between 2020 and 2026 were identified through a structured search of the Scopus database. A total of 1,331 records were screened, and 53 studies were included based on predefined inclusion and exclusion criteria. The findings were systematically categorized according to major immunopathological mechanisms.

**Results:**

The included studies indicate that endothelial dysfunction (24.5%) and cytokine dysregulation (18.9%) are the most frequently reported mechanisms in COVID-19 immunopathogenesis. Autoimmune responses (15.1%), complement activation (17%), and immune complex–mediated inflammation also play significant roles. Elevated levels of proinflammatory cytokines, lymphopenia, and markers of vascular injury were consistently associated with increased disease severity and mortality. In addition, persistent immunological alterations and autoantibody production were observed in a subset of patients during the post-COVID period.

**Conclusion:**

COVID-19 pathogenesis is driven by complex and interconnected immunopathological mechanisms rather than viral effects alone. Hyperinflammation, endothelial dysfunction, complement activation, and autoimmune processes are key determinants of disease severity and clinical outcomes. These findings underscore the importance of targeted immunomodulatory strategies and provide a comprehensive framework for future research.

## 1 Introduction

Severe acute respiratory syndrome coronavirus 2 (SARS-CoV-2), since its emergence in late 2019, rapidly led to a global pandemic and has caused significant morbidity and mortality worldwide through the clinical condition known as Coronavirus Disease 2019 (COVID-19). Initially described primarily as a respiratory infection, COVID-19 has subsequently been recognized as a complex and systemic disease capable of affecting multiple organs and systems (1, 2). The wide clinical spectrum of the disease—ranging from asymptomatic infection to severe manifestations such as acute respiratory distress syndrome, multiple organ failure, and death—has demonstrated that not only viral replication but also the nature of the host immune response plays a decisive role in disease pathogenesis (1–3).

Although the immune response developed during SARS-CoV-2 infection is essential for viral clearance, it has been shown that, in some cases, dysregulated and excessive immune activation can lead to tissue damage and increased disease severity (2, 4). In particular, the hyperinflammatory response observed in severe COVID-19 cases is characterized by increased production of cytokines and chemokines, activation of the complement system, endothelial dysfunction, and coagulopathy (3–5). This immunopathogenesis is not limited to the primary site of infection but can affect multiple organs, including the cardiovascular system, kidneys, nervous system, and hematological system (4, 6). Therefore, detailed investigation of immunopathological mechanisms is of critical importance for understanding COVID-19 pathogenesis.

Studies have shown that among the immunopathological alterations occurring during COVID-19, hyperinflammatory responses described as cytokine storm, complement-mediated inflammation, endothelial injury, and thromboinflammatory processes are particularly prominent (3–6). In addition, it has been reported that the development of autoantibodies during and after infection, along with molecular mimicry mechanisms and disruption of immune tolerance, may trigger various autoimmune diseases (7, 8). It has also been suggested that antibody responses against SARS-CoV-2 antigens may, in some cases, result in the formation of immune complexes, which can contribute to tissue damage through complement activation and vascular inflammation (5, 9). Consequently, COVID-19 is increasingly regarded not only as an infectious disease but also as a systemic condition with significant immunopathological components (2, 3).

In addition to the infection caused by SARS-CoV-2, it has been suggested that large-scale vaccination programs developed during the pandemic may, albeit rarely, lead to immune-mediated reactions (8, 10). Autoimmune phenomena and immunological changes reported following COVID-19 vaccination have highlighted the need for a better understanding of the immunopathological processes associated with both infection and immunization (8, 10).

The aim of this systematic review is to comprehensively evaluate the immunopathological mechanisms that occur during SARS-CoV-2 infection or immunization in light of the current literature, using a holistic approach. Key immunopathological processes—including cytokine storm and hyperinflammatory responses, endothelial injury, complement activation, autoimmunity, and immune complex–mediated reactions—are addressed, and potential immunological changes associated with COVID-19 vaccines are also examined. In this context, the study aims to systematically elucidate the multifaceted immunological mechanisms involved in COVID-19 pathogenesis and to provide a scientific framework for future experimental and clinical research.

## 2 Materials and methods

This study is a systematic literature review conducted to evaluate the immunopathological mechanisms that arise during SARS-CoV-2 infection and the immunological effects associated with COVID-19 vaccines. The study process included defining the research question, selecting appropriate databases, developing a search strategy, screening studies, and assessing eligibility. The systematic review process was carried out in accordance with the PRISMA (Preferred Reporting Items for Systematic Reviews and Meta-Analyses) guidelines. The literature search was performed in the Scopus database. As a result of the search using the specified keywords, 1,331 records were identified in the Scopus database. The screening process was conducted to identify up-to-date studies related to the immunopathogenesis of SARS-CoV-2 infection, and English-language research articles published between 2020 and 2026 were included. The studies obtained from the databases were first evaluated at the title and abstract level. Duplicate studies were removed by comparison across databases. The full texts of studies that passed the preliminary screening were examined and evaluated according to predefined inclusion and exclusion criteria. The inclusion and exclusion criteria are presented in Table 1.

**Table 1:** Inclusion and exclusion criteria for the systematic review.

Criterion	Inclusion	Exclusion
Publication year	Studies published between 2020–2026	Studies published before 2020
Language	English	Languages other than English
Study type	Original human studies, including prospective and retrospective cohort studies, case–control studies, cross-sectional studies, and longitudinal observational analyses investigating SARS-CoV-2 immunopathogenesis	Randomized controlled trials were not specifically targeted; editorials, commentaries, conference abstracts, and case reports were excluded
Study model	Human studies	Histological or molecular studies, animal experiments, in vitro studies, and studies not explaining immunological mechanisms
Full-text access	Studies with accessible full text	Studies without available full text

**Figure 1:**
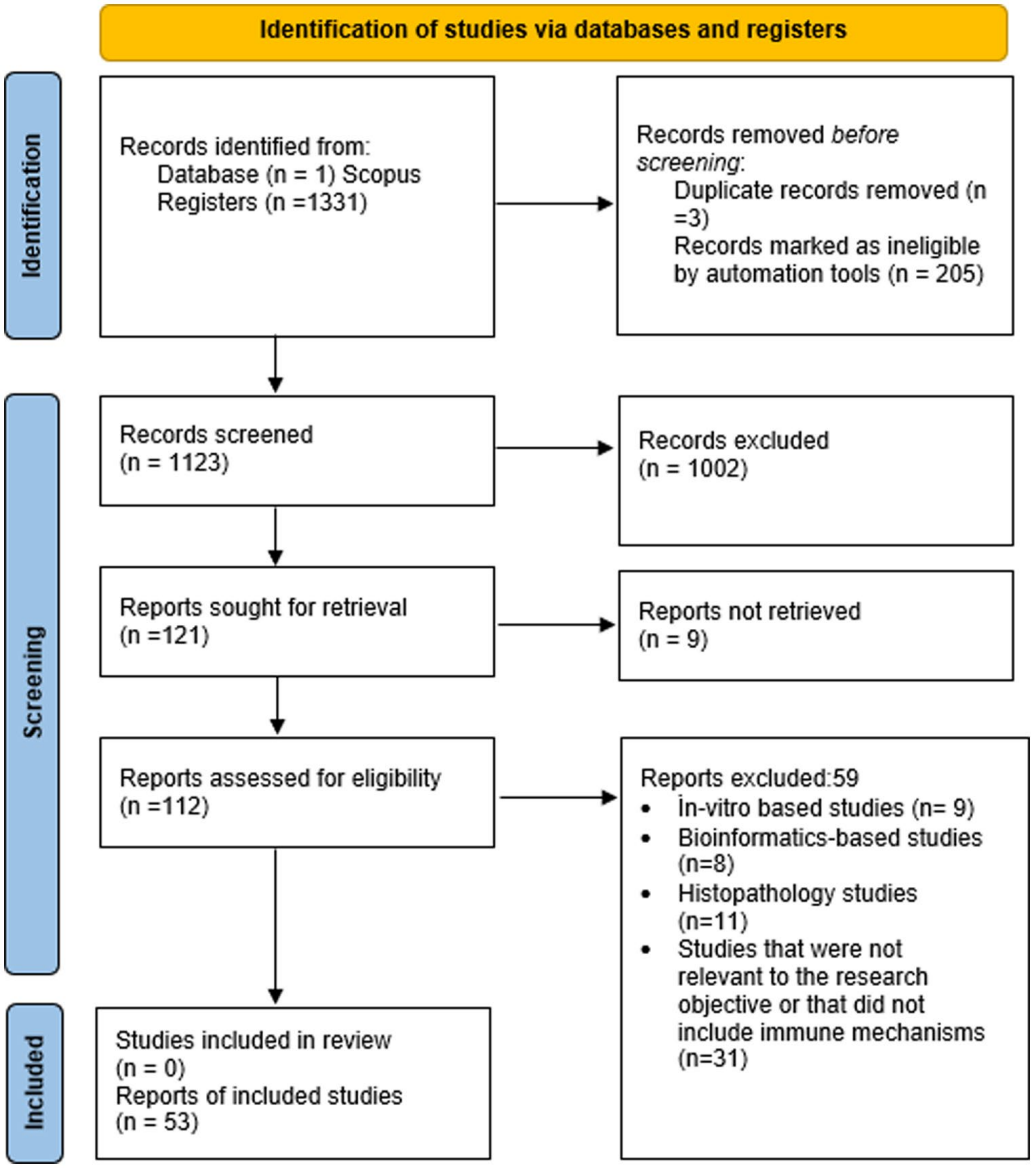
PRISMA diagram.

As a result of applying the exclusion criteria and conducting full-text reviews, a total of 53 studies were included in the systematic review and evaluated. All stages of the study selection process were illustrated using a PRISMA flow diagram (Figure 1).

The following search strategy was applied in the Scopus database:

TITLE-ABS-KEY (“SARS-CoV-2”) AND TITLE-ABS-KEY (immunopathogenesis OR “cytokine storm” OR autoimmunity OR complement OR endothelial) AND TITLE-ABS-KEY (mechanism OR pathogenesis) AND PUBYEAR > 2019 AND DOCTYPE (ar) AND LANGUAGE (English) AND NOT TITLE-ABS-KEY (mouse OR mice OR rat OR murine OR animal OR rabbit OR macaque OR “*in vitro*”) AND (LIMIT-TO (SUBJAREA, “MEDI”) OR LIMIT-TO (SUBJAREA, “IMMU”) OR LIMIT-TO (SUBJAREA, “MULT”)) AND (LIMIT-TO (SRCTYPE, “j”)).

The findings obtained from the literature were classified according to the main mechanisms associated with the immunopathogenesis of SARS-CoV-2 infection. The distribution of immunopathological mechanisms in the included studies is presented in Table 2. Among the 53 studies included in this systematic review, endothelial dysfunction and vascular damage were the most frequently investigated mechanisms (*n* = 13, 24.5%), followed by cytokine dysregulation and hyperinflammatory responses (*n* = 10, 18.9%). Autoimmune mechanisms and autoantibody formation were reported in eight studies (15.1%), while complement activation was identified in nine studies (17%). A smaller number of studies investigated immune complex-mediated inflammatory processes (*n* = 5, 9.4%).

**Table 2:** Immunopathological mechanisms identified in included studies*.

Immunopathological mechanism	Number of studies	Percentage (%)
Hyperinflammation/Cytokine dysregulation	10	18.9
Endothelial dysfunction/Vascular injury	13	24.5
Complement system activation	9	17.0
Autoantibody formation/Autoimmunity	8	15.1
Immune complex–mediated inflammation	5	9.4
Vaccine-related immune responses	8	15.1
Total	53	100

* Percentages indicate the proportion of included studies addressing each immunopathological mechanism.

## 3 Results

### 3.1 Hyperinflammation and cytokine dysregulation

A substantial proportion of the included studies demonstrate the central role of the hyperinflammatory response in the pathogenesis of COVID-19. In the study by Sims et al. (11), it was shown that increasing disease severity was associated with significant elevations in inflammatory mediators such as IFN-*γ*, IL-1RA, IL-6, IL-10, MCP-1, MCP-2, MCP-3, CXCL9, and CXCL10. These changes were also found to occur concomitantly with markers of vascular endothelial damage (11). Similarly, a large cohort study reported that IL-6, CXCL10, and particularly GM-CSF levels increased progressively with disease severity (12). Furthermore, this inflammatory profile was observed to be associated with markers of endothelial injury and thrombosis (12).

Clinical studies evaluating the prognostic significance of cytokine responses have reported comparable findings. In the study conducted by Sayah et al. (13), IL-6, procalcitonin, and the neutrophil-to-lymphocyte ratio were shown to have high predictive value in distinguishing severe and fatal forms of COVID-19. Notably, IL-6 levels were strongly associated with both disease severity and mortality (13). Moreover, distinct from the classical “cytokine storm” model, a unique inflammatory profile dominated by chemokines and growth factors has been identified in severe COVID-19, with increases in MMP-1 and VEGF-A indicating endothelial hyperactivation associated with disease severity (14).

### 3.2 Endothelial dysfunction and vascular damage

The included studies indicate that endothelial dysfunction and vascular injury play a central role in COVID-19 pathogenesis. Analyses conducted in intensive care patients have demonstrated that endothelial markers such as von Willebrand factor (VWF), PAI-1, syndecan-1, and soluble thrombomodulin are strongly associated with organ failure and thrombotic complications (15). In particular, imbalance in the VWF–ADAMTS13 axis has emerged as a key mechanism underlying COVID-19-associated microvascular thrombosis (16). Additionally, persistently elevated levels of VWF propeptide suggest ongoing endothelial activation even in the post-infectious period (17).

Classical markers of endothelial activation (ICAM-1, VCAM-1, E-selectin) and impairments in microvascular reactivity have also been found to be markedly increased in severe disease, suggesting that SARS-CoV-2 may exert direct and specific effects on the endothelium (18, 19).

Importantly, endothelial damage is not confined to the acute phase but persists into the post-acute period. Long COVID studies have reported that sustained increases in VWF, factor VIII, and thrombin generation are associated with persistent endotheliopathy and inversely correlated with functional capacity (20, 21). Furthermore, endothelial glycocalyx damage and IL-6-mediated signaling have been shown to promote a proinflammatory and procoagulant endothelial phenotype (22, 23).

### 3.3 Complement system activation

Complement system activation represents one of the key mechanisms linking inflammation, endothelial injury, and thrombosis in the immunopathogenesis of COVID-19. In SARS-CoV-2 infection, complement activation occurs via both classical and alternative pathways, with the classical pathway being particularly activated through immune complexes (24).

Clinical studies have demonstrated that complement activation markers are significantly elevated in COVID-19 patients compared to influenza and other causes of respiratory failure, with alternative pathway activation being particularly associated with endothelial damage and hypercoagulability (25). Additionally, C3 consumption and the C3a/C3 ratio have been reported to correlate strongly with in-hospital mortality (26).

Further studies have shown that SARS-CoV-2 proteins may suppress complement regulatory proteins in endothelial cells, thereby enhancing complement-mediated cytotoxicity and contributing to thrombotic microangiopathy (27). Similarly, autoantibodies directed against ACE2 may exacerbate vascular damage via complement activation (28). Moreover, the association between mannose-binding lectin (MBL) levels and thromboembolic complications suggests that the lectin pathway may also contribute to coagulopathy (29). Pediatric studies have likewise demonstrated increased levels of complement components such as C1q, C3, and C5a in COVID-19 cases (30).

### 3.4 Autoantibody formation and autoimmune mechanisms

It has been demonstrated that SARS-CoV-2 infection can trigger limited yet distinct tissue-specific autoantibody responses, which are particularly pronounced in severe disease (31). In this context, IgM autoantibodies directed against the ACE2 receptor have been shown to contribute to endothelial dysfunction via complement activation, while anti–type I interferon autoantibodies may increase the risk of thrombotic complications (28, 32).

On the other hand, some studies have reported that certain autoantibodies may persist into the convalescent phase; however, no significant increase in overt autoimmune disease has been observed in individuals with mild to moderate infection (33, 34). Nevertheless, the presence of autoantibodies associated with neurological and vascular sequelae, along with increased risk of autoimmune diseases observed in large cohort studies, supports the notion that SARS-CoV-2 may trigger autoimmune processes in specific patient populations (e.g., individuals with neurological post-COVID manifestations, severe COVID-19, or a predisposition to autoimmune diseases) (35, 36).

### 3.5 Immune complex-mediated inflammation

Although investigated in a relatively limited number of studies, immune complex–mediated inflammation has emerged as an important mechanism associated with complement activation, particularly in severe COVID-19. Increased levels of circulating immune complexes have been observed in COVID-19 patients, correlating with C1q levels and IgG responses (26). This finding suggests activation of the classical complement pathway via immune complexes (26).

Additionally, IgM autoantibodies against ACE2 have been shown to impair endothelial function through interactions with the complement system (28), while the presence of intrathecal antibodies in neurological post-COVID cases has been associated with neuroaxonal damage (35). These findings indicate that immune complexes may represent a complementary inflammatory mechanism contributing to vascular and neurological complications in COVID-19.

### 3.6 Vaccine-related immune responses

Evaluation of immunological changes following COVID-19 vaccination has shown that, particularly after adenoviral vector vaccines, there may be increases in inflammation, platelet activation, and thrombin generation, which could be associated with rare complications such as vaccine-induced immune thrombotic thrombocytopenia (VITT) (37). Additionally, although *de novo* autoantibody development has been observed in some individuals following mRNA vaccination, these changes generally do not reach the level of clinically significant autoimmune disease (38).

Importantly, the protective immunological effects of vaccination have been clearly demonstrated. Vaccinated individuals exhibit reduced inflammatory protein signatures and suppression of pulmonary fibrin deposition (39). Furthermore, inflammatory responses observed in cardiac tissue following infection have been shown to be more controlled in vaccinated individuals through distinct immunological mechanisms (40).

## 4 Discussion

This systematic review evaluated studies in the current literature on the immunopathogenesis of SARS-CoV-2 infection and demonstrated that hyperinflammation, endothelial dysfunction, complement system activation, autoantibody formation, and immune complex–mediated inflammatory processes interact and collectively contribute to the pathogenesis of COVID-19. Most of the reviewed studies indicate that COVID-19 is a highly complex disease that cannot be explained solely by viral replication, and that dysregulation of the host immune response plays a critical role in determining disease severity (1–4).

One of the most prominent immunopathological mechanisms observed in severe COVID-19 is hyperinflammation and the cytokine storm. Elevated levels of proinflammatory cytokines such as IL-6, IL-1β, and TNF-*α* are strongly associated with disease severity and mortality (3–5, 41). It should be noted, however, that no single inflammatory biomarker was assessed consistently across all included studies. This heterogeneity likely reflects differences in study design, patient populations, disease severity, and laboratory methodologies. Nevertheless, despite this variability, the overall findings converge in demonstrating that dysregulated inflammatory responses involving cytokines, chemokines, and growth factors are closely linked to adverse clinical outcomes. Cytokine-mediated inflammation not only amplifies the acute inflammatory response but also contributes to multi-organ damage through activation of the vascular endothelium and stimulation of the coagulation system. Furthermore, impairments in early antiviral defense mechanisms—such as delayed or inadequate interferon responses—have been reported to facilitate increased viral load and uncontrolled inflammation (2, 3). Taken together, these findings suggest that the inflammatory response in SARS-CoV-2 infection is not limited to elevated cytokine levels, but rather represents a multilayered immune dysregulation involving chemokines, growth factors, and endothelium-associated inflammatory mediators. Among the various biomarkers evaluated, IL-6 emerged as the most consistently reported marker associated with disease severity, while the IL-6 and GM-CSF axis appears to be a key component linking systemic inflammation to vascular injury in severe disease (41–43).

The studies included in this review clearly demonstrate the central role of the vascular system in COVID-19 pathogenesis. The ability of SARS-CoV-2 to infect endothelial cells via ACE2 receptors leads to endothelial dysfunction and the development of thromboinflammatory processes (41, 42). Endothelial injury promotes the upregulation of adhesion molecules and procoagulant factors, facilitating microvascular thrombosis and impairing organ perfusion. The association of markers such as von Willebrand factor, syndecan-1, and PAI-1 with severe disease and thrombotic complications supports this mechanism (43). Moreover, the persistence of endothelial activation and microvascular alterations in the post-acute phase suggests that vascular pathology may also contribute to long-term sequelae (44, 45).

Complement system activation emerges as a key mechanism that reinforces the interplay between inflammation and thrombosis. Increased levels of anaphylatoxins such as C3a and C5a enhance inflammatory cell activation, while the membrane attack complex exacerbates endothelial injury (46, 47). Collectively, the available evidence indicates that the complement system constitutes a central pathophysiological axis integrating inflammation, endothelial dysfunction, and immunothrombosis in COVID-19.

The relationship between COVID-19 and autoimmune mechanisms has also been increasingly investigated. The intense inflammatory milieu during SARS-CoV-2 infection, along with polyclonal B-cell activation and disruption of immune tolerance, may predispose to autoantibody formation (48, 49). Several studies have reported the presence of antinuclear antibodies, antiphospholipid antibodies, and autoantibodies against type I interferons in patients with COVID-19 (49, 50). Notably, the higher prevalence of anti-interferon autoantibodies in severe cases suggests a potential role for autoimmune processes in impairing antiviral immune responses (49). Molecular mimicry has also been proposed as an important pathogenic mechanism in the development of autoimmunity. A bioinformatics-based study identified significant peptide homology between the SARS-CoV-2 spike protein and various endocrine autoantigens. In this analysis, the spike protein was segmented into pentapeptides and compared with thyroid peroxidase, thyroglobulin, TSH receptor, pancreatic *β*-cell autoantigens, and adrenal enzymes, revealing a total of 14 shared pentapeptide sequences. A substantial proportion of these sequences were located within immunologically active epitope regions, suggesting that SARS-CoV-2 infection may trigger endocrine autoimmune diseases—such as autoimmune thyroid disorders, type 1 diabetes, and autoimmune adrenal diseases—via molecular mimicry mechanisms (51).

The included studies differed substantially in terms of timing, population, and immunological endpoints. While cytokine dysregulation and hyperinflammation were mainly investigated during the acute phase of severe COVID-19, autoantibody formation and autoimmune manifestations were more commonly evaluated in post-acute or convalescent periods. Therefore, the available evidence does not permit a precise estimation of the time interval between acute hyperinflammation and subsequent autoimmune phenomena, but supports the concept that these mechanisms may occur at different stages of SARS-CoV-2-related immune dysregulation.

Although less extensively studied, immune complex–mediated inflammation is considered a complementary mechanism in COVID-19 pathogenesis, particularly in relation to complement activation. The deposition of antigen–antibody complexes in the vascular endothelium and subsequent activation of the classical complement pathway may lead to microvascular inflammation and tissue damage (5, 52). Indeed, the prominence of vascular inflammation and endothelial injury in COVID-19–associated dermatological manifestations supports this mechanism (53).

In addition to findings from the literature, preliminary observations from thesis studies conducted at our center have provided insights into post-COVID immunological alterations. Evaluations of individuals with different vaccination patterns revealed low-titer positivity of autoantibodies such as ANA, ANCA, and anti-CCP in some cases; however, this was not directly associated with clinically significant autoimmune disease development (54). Furthermore, increased levels of complement activation markers (C3a, C5a) along with circulating immune complexes were observed in individuals with prior COVID-19 infection (55). Nevertheless, these findings should be interpreted with caution due to limited sample size and lack of independent validation.

When the effects of COVID-19 vaccines on the immune system are considered, the available evidence should be interpreted in light of the limited nature of the published data. Most reports describing vaccine-associated immunological events consist of case reports or small observational studies and therefore do not allow reliable estimation of the frequency of individual immune dysregulations. Nevertheless, current evidence indicates that, despite rare immune-mediated reactions such as vaccine-induced immune thrombotic thrombocytopenia and occasional *de novo* autoantibody formation, COVID-19 vaccines are generally safe and do not confer a clinically meaningful increase in the risk of autoimmune disease development (56, 57). Importantly, the overall protective effects of vaccination are well established. Clinical studies have shown that vaccinated individuals experience significantly lower mortality rates (12.5% vs. 21.8%) and markedly higher recovery rates compared with unvaccinated patients (58). Similarly, full vaccination has been associated with reduced mortality and lower rates of intensive care unit admission (59). These findings demonstrate that, although rare immune-mediated adverse events have been reported following vaccination, such events occur far less frequently than the immunopathological complications associated with natural SARS-CoV-2 infection. In contrast, SARS-CoV-2 infection itself appears to induce substantially more pronounced and clinically significant immunopathological effects.

A limitation of this study is that the literature search was conducted exclusively in the Scopus database. Although Scopus provides broad coverage of high-impact biomedical and multidisciplinary journals, relevant studies indexed solely in other databases may not have been captured. In addition, only English-language research articles were included, and studies published in other languages were excluded. Furthermore, methodological heterogeneity and differences in sample sizes among the included studies may limit the direct comparability of some findings. Despite these limitations, this study provides a significant contribution by systematically evaluating the current literature on the immunopathogenesis of SARS-CoV-2 infection.

In conclusion, this systematic review demonstrates that the pathogenesis of COVID-19 is shaped not merely by the direct effects of viral replication but by the interaction of multiple immunopathological mechanisms. The development of immunopathological complications following SARS-CoV-2 infection appears to be influenced by multiple host-related factors, including age, underlying comorbidities, genetic predisposition, and potentially environmental determinants such as diet, gut microbiome composition, and socioeconomic conditions. These factors may modulate immune responses and help explain why severe immune dysregulation develops in only a subset of infected individuals. Importantly, the immunopathological mechanisms described in this review rarely occur in isolation; rather, hyperinflammation, endothelial dysfunction, complement activation, and autoimmune responses frequently coexist and interact, collectively shaping the clinical heterogeneity of COVID-19. Based on the findings of this systematic review, we propose that hyperinflammation and cytokine dysregulation represent the principal initiating mechanisms in COVID-19 immunopathogenesis. Excessive production of proinflammatory cytokines and chemokines may trigger and amplify a cascade of interconnected processes, including endothelial activation, complement system activation, immunothrombosis, autoantibody formation, and immune complex–mediated tissue injury. These mechanisms collectively contribute to microvascular dysfunction, multi-organ damage, and both acute and post-acute clinical manifestations. While endothelial dysfunction and complement activation emerge as major drivers of thromboinflammatory processes, autoantibody formation and immune complex–mediated mechanisms may contribute to clinical complications in specific patient populations. In particular, endothelial injury and immunothrombosis appear to play critical roles in the development of severe disease and organ dysfunction. A deeper understanding of the immunopathogenesis of SARS-CoV-2 infection is essential for predicting disease course and developing targeted therapeutic strategies. Future large-scale clinical and molecular studies are expected to further elucidate the interactions among inflammation, complement activation, and autoimmune mechanisms.

## Data Availability

The original contributions presented in the study are included in the article/supplementary material, further inquiries can be directed to the corresponding author.
